# Research progress on *Bergenia purpurascens*: a narrative review

**DOI:** 10.3389/fphar.2026.1863591

**Published:** 2026-09-30

**Authors:** Li Chang-xi, Xu Xiang, Fu Ying, Xie Zongyi, Li Lin-zhe, Yu Jie, Li Xue-fang

**Affiliations:** 1 School of Chinese Materia Medica, Yunnan University of Traditional Chinese Medicine, Kunming, Yunnan, China; 2 College of Chinese Materia Medica and Yunnan Key Laboratory of Southern Medicine Utilization, Yunnan University of Traditional Chinese Medicine, Kunming, Yunnan, China

**Keywords:** *Bergenia purpurascens*, bergenin, chemical metabolite, pharmacological action, research progress

## Abstract

*Bergenia purpurascens* (Hook.f.et Thoms.) Engl., a perennial herb belonging to the genus *Bergenia* (family Saxifragaceae), is primarily distributed in southwestern China and mountainous regions of Nepal, Bhutan, and northeastern India, where it has a long history of medicinal use. A systematic retrieval of relevant literature was conducted on PubMed, Scopus, CNKI, and Web of Science using “*Bergenia purpurascens*” and “*Bergenia*” as primary keywords, supplemented by “chemical metabolite,” “pharmacological action,” and “resource distribution” as search terms. This review systematically summarizes the research progress on the botanical characteristics, resource distribution, medicinal parts, chemical metabolites, pharmacological activity, toxicology, and modern applications of *B. purpurascens.* Its primary active metabolites include phenolics, including bergenin, gallic acid, and arbutin, which exhibit expectorant, antitussive, anti-inflammatory, hepatoprotective, antitumor, and neuroprotective activities. Recent studies elucidating the bergenin biosynthetic pathway provide a basis for industrial production. Additionally, *B. purpurascens* shows broad development potential in pharmaceuticals, health products, cosmetics, and landscaping. This review provides a scientific basis for further research and comprehensive utilization of *B. purpurascens*.

## 1 Introduction

*Bergenia* *purpurascens* (Hook.f.et Thoms.) Engl. is a species of the genus *Bergenia* (family Saxifragaceae). It is characterized as slightly aromatic, with a bitter and astringent taste, and is neutral in property, and considered to act on the lung, liver, and spleen meridians. It is used to treat diarrhea through astringent effects, relieve cough and bleeding, relax tendons, and activate collaterals and is applied clinically for diarrhea, dysentery, internal and external traumatic bleeding, pulmonary tuberculosis-related cough, tracheitis, and traumatic injuries (National Pharmacopoeia Committee, 2025). Its primary active metabolite, bergenin, has good antitussive and expectorant effects and is widely used worldwide (Liu et al., 2023). As a commonly used medicinal material in traditional Chinese medicine and in Tibetan, Naxi, Yao, Uyghur, and other ethnomedical systems, *B. purpurascens* has a long history of application and extensive empirical use. Globally, the genus comprises 10 species, seven of which occur in China; among them, *B. emeiensis*, *B. scopulosa*, and *B. tianquanensis* are endemic. These species are mainly distributed in high-altitude, humid rock crevices and miscellaneous forests of southwestern and northwestern China (Yang et al., 2023), thereby conferring medicinal, edible, ornamental, and ecological value. Therefore, in addition to focusing on *B. purpurascens*, which possesses the highest medicinal value, this review presents a brief comparative discussion of other congeneric species to provide complete genus-level background information and establish a foundation for the rational analysis of the pharmacological effects of plants in this genus.

The primary metabolites of *B. purpurascens* have been reported to be phenolic metabolites, including bergenin, gallic acid, and arbutin, with bergenin as the main active metabolite (Xia et al., 2017). Pharmacological studies have demonstrated various bergenin biological activities, including expectorant, antitussive, anti-inflammatory, hepatoprotective, antitumor, neuroprotective, antidiabetic, and hypolipidemic effects (Salimo et al., 2023). Although substantial progress has been achieved in resource protection, metabolite analysis, pharmacological mechanisms, and product development, challenges remain, including limited systematic toxicology data, insufficient product development depth, and unsustainable use of wild resources. This review systematically summarizes research progress in the botanical characteristics, resource distribution, cultivation and harvesting, chemical metabolite, pharmacological activity, quality control, and modern applications of *B. purpurascens* and further outlines existing achievements and shortcomings to support resource protection, in-depth development, and scientific utilization.

A systematic literature search was conducted using the keywords “*B. purpurascens*” and “*Bergenia*.” Four major databases, namely, PubMed, CNKI, Web of Science, and Scopus, were searched, yielding a total of 3,003 records. After removing 1,895 duplicate entries, 1,108 independent studies were retained for screening titles and abstracts. According to the exclusion criteria, 142 review articles were excluded because of incomplete reporting (e.g., lack of key methodological information), 452 records were excluded because their titles or abstracts were deemed irrelevant, 360 studies were excluded because they were unrelated to the research objectives, and 66 studies were excluded for other reasons. Finally, the final analysis included 88 articles that met the predefined inclusion and exclusion criteria (Figure 1).

**FIGURE 1:**
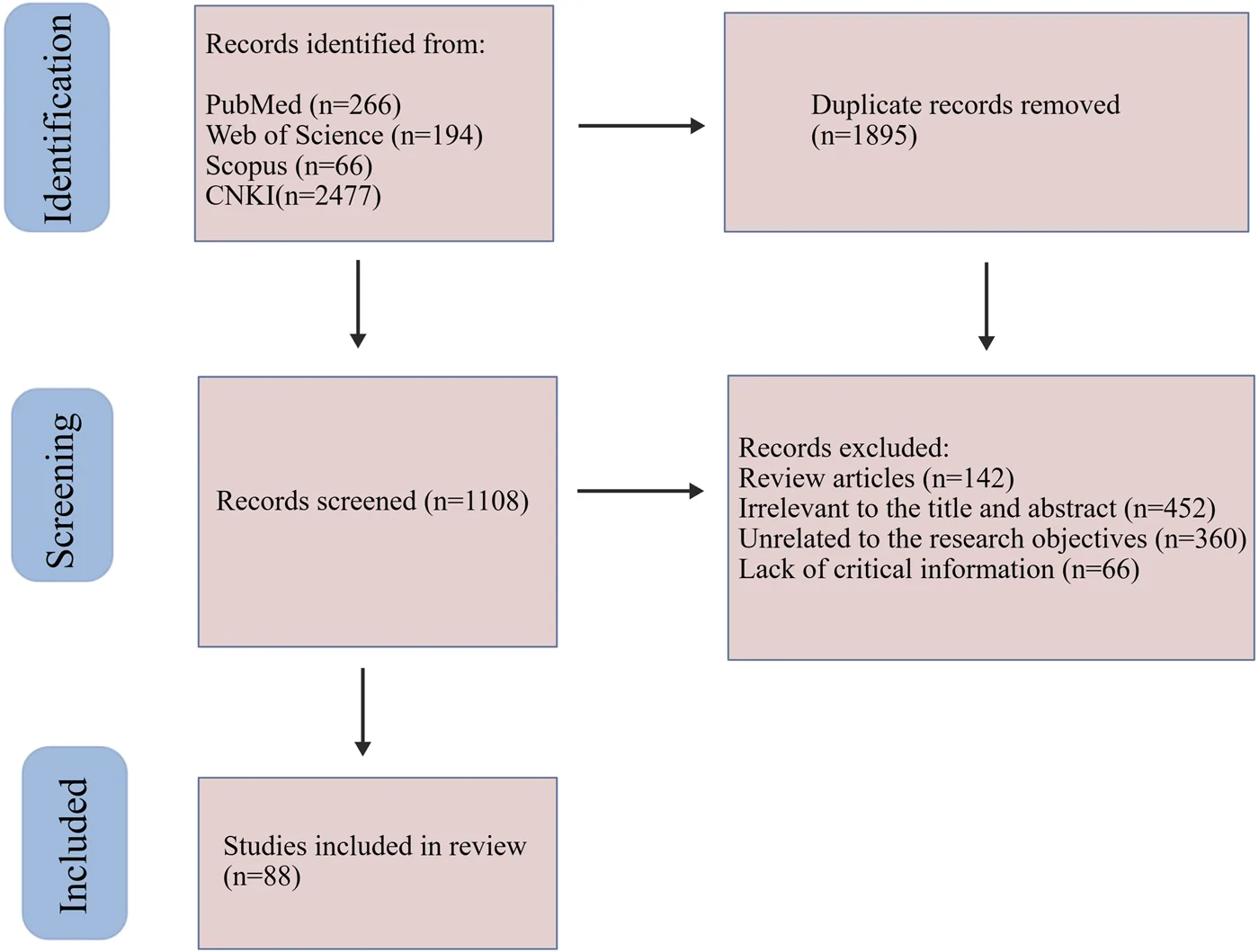
PRISMA flowchart for Article selection.

## 2 Historical evolution of *Bergenia purpurascens*

The name “Yan Bai Cai” first appeared in Qing Dynasty local herbal literature, named because it “grows on rocks” and has cabbage-like leaves. In the Illustrated Catalogue of Plants, it is recorded as “Dai Bai Cai” and “Ai Bai Cai,” with noted efficacy in treating hemoptysis (Wu and Lu, 1848). The Classification of Herbal Properties describes its ability to resolve phlegm and stop cough, and treat internal injury-related hemoptysis, asthma, and stranguria (Anonymous, 1906). Qing Dynasty medical texts also recorded its use for febrile diseases and bleeding syndromes, indicating its early clinical applications.

The modern botanical classification of *B. purpurascens* began in the mid-19th century. In 1857, British botanists Hooker f. and Thomson described the species as *Saxifraga purpurascens* (Hooker and Thomson, 1857). In 1868, German botanist Engler reassigned it to the genus *Bergenia*, establishing the accepted scientific name *B. purpurascens* (Hook. f. et Thomson) Engl (Engler, 1868), which is now recognized in international databases such as The Plant List and Kew Science.

In the mid-to-late 20th century, advances in natural medicinal chemistry facilitated the identification of its active metabolites, including bergenin, arbutin, and gallic acid, with bergenin as the characteristic and primary pharmacologically active metabolite (Xia et al., 2017). Bergenin, a cyclized aromatic C-glycoside first isolated from *Bergenia* species, accounts for its antitussive and expectorant effects (Salimo et al., 2023). Consequently, *B. purpurascens* was included in the Pharmacopoeia of the People’s Republic of China as an antitussive medicine. The development and clinical application of Compound Bergenin Tablets for chronic bronchitis marked its transition from a traditional herbal medicine to a modern pharmaceutical.

Since the 21st century, the applications of *B. purpurascens* have expanded further. Its cold tolerance, drought resistance, evergreen nature, and bright flowers support its widespread use in rock gardens, understory greening, and indoor cultivation (An, 2023). In cosmetics, its ultraviolet ray-blocking properties have enabled the development of sunscreen products. Additionally, its tender leaves are consumed as vegetables or stir-fried to make tea, making it an edible resource.

## 3 Resources and distribution of Bergenia species

### 3.1 Overview of *Bergenia purpurascens* resources

The rhizome of B. purpurascens (Hook.f.et Thoms.) Engl. [Saxifragaceae (Figure 2); is commonly called Dai Bai Cai, Ai Bai Cai, Yan Bi Cai, Yan Chang Pu, Hong Duan Zi, Guan Yin Lian, Zi Geng, Yan Qi, Mi Hei Zhe, Ai Su Meng, Ai Chang Pu, Ha Dong, and You Yue Bai. Taxonomically, it belongs to the genus *Bergenia* within Saxifragaceae. The species typically inhabits dark, humid environments such as miscellaneous forests and rock crevices at altitudes of 2,700–4,800 m. In China, it is mainly distributed in southern and eastern Tibet, southwestern Sichuan, and northern Yunnan and is also distributed in northern Myanmar, northeastern India, northern Bhutan, and Nepal. *Bergenia purpurascens* is a perennial herb characterized by a thick purple-red rhizome, a capitate stigma, short internodes, and erect capsules bearing many seeds. Its flowering and fruiting period extends from May to October (Delectis Florae Reipublicae Popularis Sinicae Agendae Academiae Sinicae Edita, 1992) (Figures 2A,B). The cultivation of *B. purpurascens* typically involves harvesting after 2 years, during which large mature plants are dug out and smaller immature ones are left behind. The rhizomes are then washed, and the withered leaves are trimmed and sun-dried or used fresh (Wu et al., 2001). It is sweet and astringent in taste, cool in nature, and acts on the lung, liver, and spleen meridians. Medicinally, it exerts nourishing and strengthening, antitussive, and hemostatic effects and is commonly used for weakness, dizziness, deficiency-related cough and asthma, fatigue-induced hemoptysis, hematemesis, stranguria, and leukorrhea.

**FIGURE 2:**
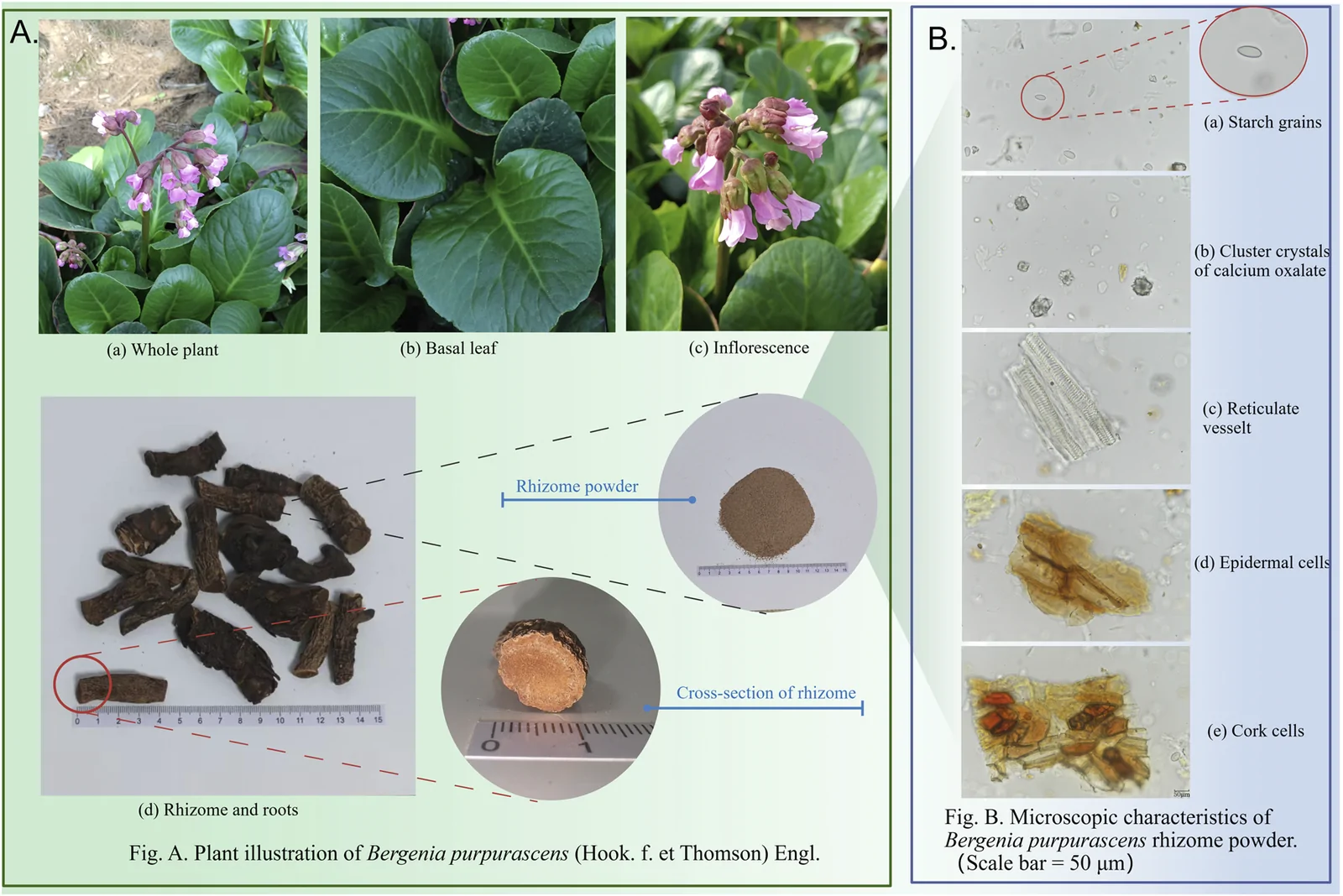
Botanical and microscopic characteristics of Bergenia purpurascens (Hook. f. et Thoms.) Engl. **(A)** Botanical and medicinal images of B. purpurascens: (a) Whole plant; (b) Basal leaf; (c) Inflorescence; (d) Rhizome and roots (including rhizome powder and cross-section of rhizome). **(B)** Microscopic characteristics of B. purpurascens rhizome powder: (a) Starch grains; (b) Cluster crystals of calcium oxalate; (c) Reticulate vessel; (d) Epidermal cells; (e) Cork cells. (Scale bar = 50 µm).

### 3.2 Medicinal uses

The medicinal parts of *B. purpurascens* vary across regions and ethnomedicinal systems (Table 1). The rhizome is primarily used for cough relief, hemostasis, anti-inflammatory analgesia, and treatment of urinary disorders, whereas the leaves are mainly used for beverages or external applications, reflecting their heat-clearing and detoxifying functions.

**TABLE 1:** Medicinal parts and main uses of *Bergenia purpurascens* in various application systems.

Application system	Region/ethnicity	Medicinal part	Main use	Specific application/preparation	References
Chinese Traditional Medicine	China	Whole plant or rhizome	Nourishing and strengthening; antitussive; hemostatic	Treats weakness and dizziness, lung deficiency cough, fatigue-induced hemoptysis, vomiting blood, stranguria, leukorrhea	—
	Chinese Herbal Classics	Rhizome	Hemostatic; nourishing	Illustrated Catalogue of Plants: “Treats vomiting blood”; Classified Herbal Medicine: “Resolves phlegm and stops cough; treats internal injury, hematemesis, wheezing, and stranguria”	—
Chinese Ethnic Medicine	Tibetan	Rhizome	Heat-clearing and detoxifying; liver and stomach regulation; antitussive	Used for pneumonia and lung deficiency cough; antidote for liver and stomach toxins; Tibetan compound formulations for respiratory inflammation (combined with *Phlomis younghusbandii*, *Artemisia scoparia*, and *Glycyrrhiza uralensis*)	—
	Naxi	Whole plant	Regulating qi; anti-inflammatory; digestive support; antipyretic	Used for nephritis, edema, and pneumonia; exhibits fever-reducing and astringent effects	—
	Yao	Whole plant	Lung-clearing; antitussive; hemostatic; anti-dysenteric	Mainly treats pulmonary tuberculosis-induced cough, hemoptysis, hematemesis, epistaxis, hematochezia, enteritis, dysentery, uterine bleeding, leukorrhea, irregular menstruation, and impetigo	—
	Uyghur	Whole plant	Antitussive; antiasthmatic	Used for bronchitis and cough	—
International Usage	India	Rhizome	Dissolving kidney and bladder stones	Prepared as Ayurvedic formulations for urinary stones	(Liu et al., 2023)
	Russia	Leaf	Healthy beverage	Leaves are commonly used to prepare traditional healthy beverages; darkened leaves are used in the Altai region, while fresh leaves are used by Buryats and Mongolians	(Liu et al., 2023)
	Nepal	Rhizome	Antitussive; antiasthmatic; anti-inflammatory	Used to treat cough and asthma, similar to TCM usage	(Liu et al., 2023)
	Bhutan	Rhizome	Hemostatic; anti-dysenteric	Used for dysentery and diarrhea	(Liu et al., 2023)
	Northern Myanmar	Rhizome	Anti-inflammatory; analgesic	Used for rheumatic pain	(Liu et al., 2023)

### 3.3 Comparison of morphological characteristics of congeneric species

The perennial species *B. purpurascens* is a herb with thick rhizomes and basal leathery leaves. However, they differ significantly in leaf morphology, hair type, and flower structure (Table 2).

**TABLE 2:** Comparison of morphological characteristics of eight *Bergenia* species.

Latin name	Root	Leaf	Flower
*Bergenia purpurascens* (Hook. f. et Thomson) Engl.	Rhizome stout, covered with scales	Basal, leathery, obovate to subelliptic; margin sinuate-dentate to subentire; base cuneate; glabrous with small glands on both sides	Corymbose panicle; pedicels and branches densely covered with long-stalked glandular hairs; sepals densely glandular abaxially; petals purple-red, broadly ovate
*Bergenia pacumbis* (Buch.-Ham. ex D. Don) C. Y. Wu et J. T. Pan	Rhizome stout, with scales and persistent petiole sheaths	Basal, leathery, orbicular to obovate; margin subentire or weakly crenate; stiff cilia present, base slightly cordate, glabrous	Corymbose panicle; pedicels and branches sparsely glandular; sepals glabrous; petals white or pinkish; suborbicular
*Bergenia scopulosa* T. P. Wang	Rhizome stout, densely covered with brown scales and persistent leaf sheaths	Basal, leathery, orbicular to broadly elliptic; margin sinuate or sinuate-dentate; base rounded, glabrous with small glands	Corymb; pedicels glabrous; hypanthium externally glabrous; sepals glabrous; petals white or slightly pinkish; elliptic to suborbicular
*Bergenia crassifolia* (L.) Fritsch	Rhizome stout, with scales and withered petiole sheaths	Basal, leathery, obovate to elliptic; margin sinuate-dentate; base cuneate; glabrous with small glands	Corymbose panicle; pedicels and branches with subsessile glandular hairs; sepals sparsely glandular abaxially; petals reddish-purple; elliptic to oblate
*Bergenia emeiensis* C. Y. Wu	Rhizome stout, with scales and persistent petiole sheaths	Basal, leathery, narrowly obovate; margin entire; base cuneate; glabrous	Corymbose panicle; pedicels and branches sparsely with subsessile glandular hairs; sepals abaxially; petals white; narrowly obovate
*Bergenia stracheyi* (Hook. f. et Thoms.) Engl.	Rhizome stout	Basal, obovate; margin serrate or biserrate with stiff cilia; base cuneate or slightly rounded, both sides glabrous	Corymbose panicle; pedicels and branches with long-stalked glandular hairs; sepals glandular abaxially; petals red; subspatulate
*Bergenia tianquanensis* J. T. Pan	Rhizome stout, dark brown, covered with scales	Basal, leathery, obovate to broadly obovate; margin toothed and serrate with dense cilia; base cuneate	Corymb; sepals glandular abaxially; petals pale red; elliptic
*Bergenia ciliata* (Haw.) Sternb.	Rhizome stout, creeping, with scars and axillary buds	Basal, fleshy, broadly obovate to elliptic; margin finely toothed or shallowly sinuate-dentate with stiff cilia	Corymbose panicle; flowers white, pinkish, or purple

### 3.4 Distribution of congeneric species resources

Globally, the genus *Bergenia* comprises 10 species, mainly distributed in northern Myanmar, Nepal, northeastern India, and northern Bhutan (Figure 3A). Pakistan, Kashmir, and India each harbor three species. In northern South Asia, representative species include *B. pacumbis*, *B. stracheyi*, and *B. purpurascens*. Sikkim and Bhutan each harbor two species, whereas northern Korea, Mongolia, and East Asia each harbor one species. China harbors seven species (Xia et al., 2017), among which *B. emeiensis*, *B. scopulosa*, and *B. tianquanensis* are endemic (Table 3).

**FIGURE 3:**
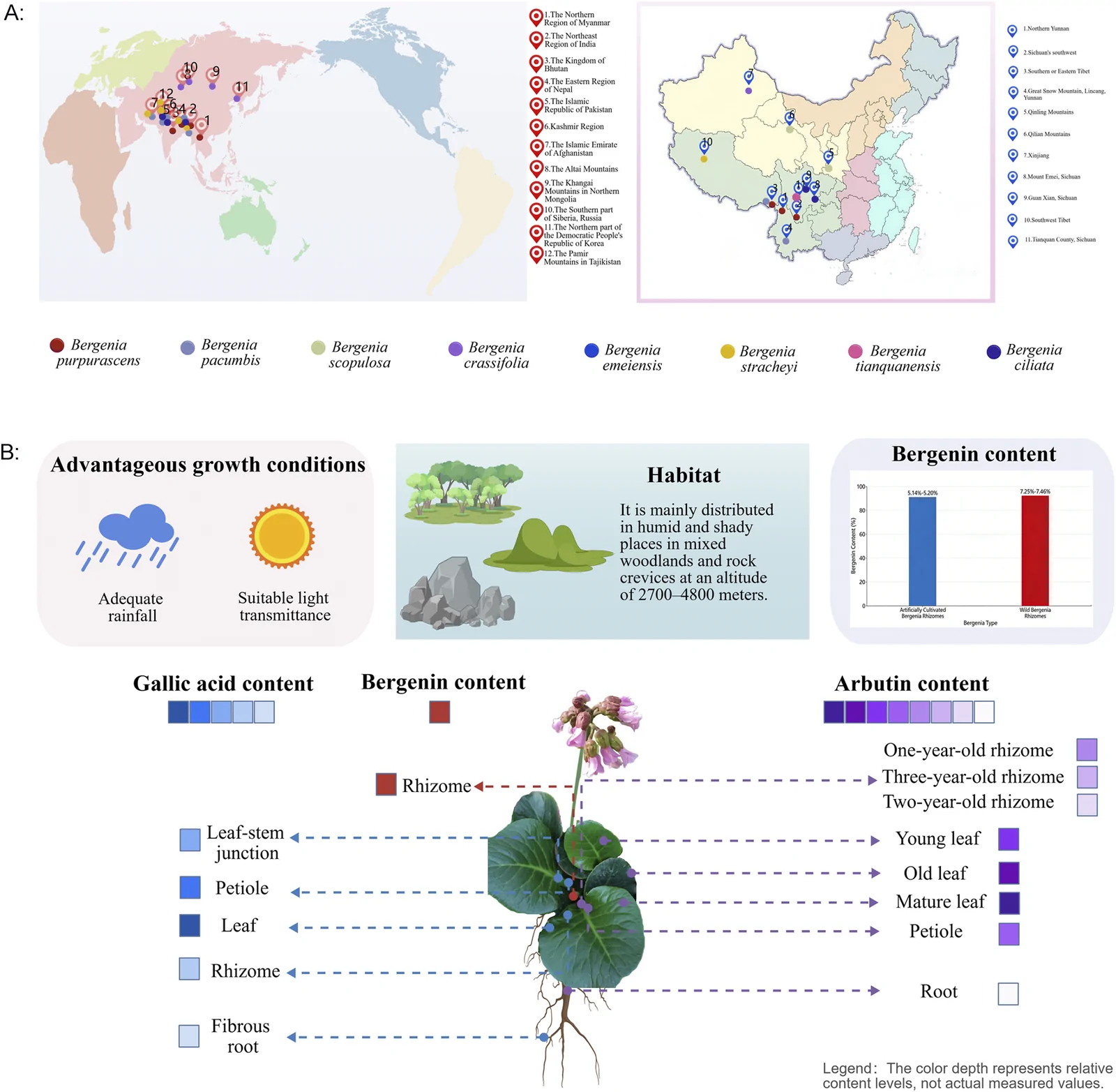
Distribution of Bergenia resources and dynamics of bergenin accumulation. **(A)** Global and Chinese distribution maps of Bergenia resources. **(B)** Diagram of growth conditions, habitat, and accumulation of active components in Bergenia.

**TABLE 3:** Domestic and international distribution of *Bergenia* species.

Latin name	Distribution in China	International distribution	References
*Bergenia purpurascens* (Hook. f. et Thomson) Engl.	Southwestern Sichuan, northern Yunnan, and southern and eastern Tibet	Northern Myanmar, northeastern India, northern Bhutan, and Nepal	(Delectis Florae Reipublicae Popularis Sinicae Agendae Academiae Sinicae Edita, 1992)
*Bergenia pacumbis* (Buch.-Ham. ex D. Don) C. Y. Wu et J. T. Pan	Yunnan (Lincang Daxue Mountain) and southern Tibet	Northeastern India, Bhutan, Nepal, Pakistan, Kashmir region, and Afghanistan	(Delectis Florae Reipublicae Popularis Sinicae Agendae Academiae Sinicae Edita, 1992)
*Bergenia scopulosa* T. P. Wang	Shaanxi (Qinling Mountains) and Qilian Mountains region	Endemic to China	(Delectis Florae Reipublicae Popularis Sinicae Agendae Academiae Sinicae Edita, 1992)
*Bergenia crassifolia* (L.) Fritsch	Xinjiang	Altai Mountains, northern Mongolia, former Soviet Union, and northern Korea	(Delectis Florae Reipublicae Popularis Sinicae Agendae Academiae Sinicae Edita, 1992)
*Bergenia emeiensis* C. Y. Wu	Sichuan (Mount Emei, Guanxian)	Endemic to China	(Delectis Florae Reipublicae Popularis Sinicae Agendae Academiae Sinicae Edita, 1992)
*Bergenia stracheyi*	Southwestern Tibet	Northern India, Nepal, western Pakistan, eastern Afghanistan, former Soviet Union (Tajikistan), and Kashmir region	(Delectis Florae Reipublicae Popularis Sinicae Agendae Academiae Sinicae Edita, 1992)
*Bergenia tianquanensis*	Sichuan (Tianquan County)	Endemic to China	(Pan, 1994)
*Bergenia ciliata*	—	Pakistan, Kashmir region, and Nepal	(Shi, 2013)
*Bergenia hissarica*	—	Former Soviet Union	(Shi, 2013)
*Bergenia ugamica*	—	Former Soviet Union	(Shi, 2013)

### 3.5 Adulterants and substitutes of *Bergenia purpurascens*

Various adulterants and substitutes for *B. purpurascens* are present in the market. Common adulterants include *Boea crassifolia*, *Corallodiscus flabellatus*, *Acorus tatarinowii*, and *Primulina eburnea*, which are frequently misused due to similar morphology and nomenclature, as well as intentional adulteration. Substitutes primarily include *Rodgersia sambucifolia*, *Rodgersia pinnata*, *Rodgersia aesculifolia*, and *Ardisia japonica*. These species contain bergenin and may serve as alternative plant sources for bergenin extraction (Table 4; Figure 4).

**TABLE 4:** Adulterants and substitutes of *Bergenia purpurascens*.

Category	Latin name	Plant source	Description	References
Adulterant	*Boea crassifolia* Hemsl.	Whole plant of *Boea crassifolia* Hemsl. (family Gesneriaceae)	In Sichuan, Guizhou markets, it is easily confused with genuine *B. purpurascens* due to similar morphology or nomenclature	(Shu and Zhang, 1991)
	*Corallodiscus flabellatus* (Craib.) Burtt.	Whole plant of *Corallodiscus flabellatus* (Craib.) Burtt (family Gesneriaceae)	Same as above, appears as adulterant in some regional markets	(Shu and Zhang, 1991)
	*Acorus tatarinowii* Schott	Dried rhizome of *Acorus tatarinowii* Schott (family Araceae)	*B. purpurascens* is a counterfeit for *A. tatarinowii*, may be adulterated by unscrupulous merchants	(Deng et al., 2024)
	*Primulina eburnea*(Hance) Yin Z.Wang	Fresh or dried rhizome or whole plant of *Primulina eburnea* (Hance) Yin Z.Wang (family Gesneriaceae)	Miao medicine “Yan Bai Cai” appears under the name *P. eburnea*, similar name but different plant, often confused	(Li et al., 2023)
Substitute	*Rodgersia sambucifolia*	Dried rhizome of *Rodgersia sambucifolia* (family Saxifragaceae)	Main raw material for bergenin extraction; can substitute *B. purpurascens* as bergenin source	(Li et al., 2009)
	*Rodgersia pinnata*	Dried rhizome of *Rodgersia pinnata* (family Saxifragaceae)	Main raw material for bergenin extraction; can substitute *B. purpurascens* as bergenin source	(Li et al., 2009)
	*Rodgersia aesculifolia*	Dried rhizome of *Rodgersia aesculifolia* (family Saxifragaceae)	Main raw material for bergenin extraction; can substitute *B. purpurascens* as bergenin source	(Li et al., 2009)
	*Ardisia japonica* (Thunb.) Blume	Dried whole plant of *Ardisia japonica* (Thunb.) Blume (family Primulaceae)	Plant resource for bergenin source, same as *B. purpurascens*	(Li et al., 2009)

**FIGURE 4:**
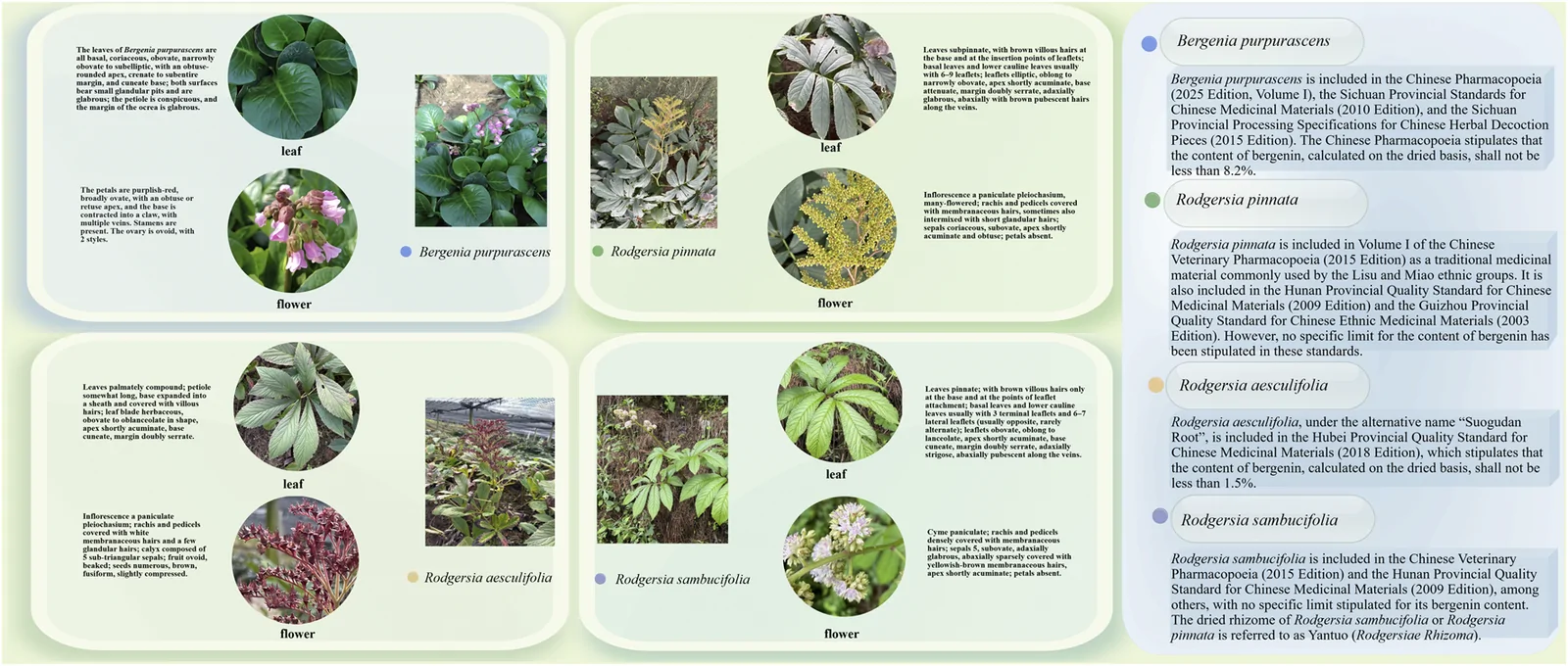
Botanical morphology of *Bergenia purpurascens* and its substitutes.

### 3.6 Cultivation and harvesting

According to the Pharmacopoeia of the People’s Republic of China (2025 Edition, Part I), the medicinal material *B. purpurascens* is derived from the dried rhizome of *B. purpurascens* (Hook.f.et Thoms.) Engl., harvested in autumn and winter, with leaf sheaths and impurities removed, and subsequently sun-dried. In recent years, many studies have systematically investigated cultivation conditions, provenance screening, correlations between agronomic traits and active metabolites, and optimal harvesting periods for different plant parts (Xia et al., 2017). Evidence indicates that sufficient rainfall promotes increased bergenin content in *Bergenia* species (Wang et al., 2017), while 60% light transmittance is most conducive for *B. tianquanensis* growth (Li et al., 2011). The Yunnan Academy of Agricultural Sciences reported that bergenin content in artificially cultivated *B. purpurascens* rhizomes (5.14%–5.20%) is slightly lower than that in wild plants (7.25%–7.46%) (Shu and Zhang, 1991), indicating both feasibility and development potential for artificial cultivation. Bergenin yield per plant is significantly positively correlated with rhizome diameter, suggesting that plant height and rhizome diameter can serve as reference traits for breeding and cultivation (Zhu et al., 2013). Additionally, the selection of harvest part and timing directly affects the yield and quality of active metabolites. One study showed that gallic acid content follows the order: leaves > petioles > stem–leaf junction > rhizome > fibrous roots, with leaves serving as the primary site of synthesis and accumulation; summer is the optimal harvest period (Liu et al., 2015). Arbutin content follows the order: mature leaves > old leaves > young leaves > petioles > one-year-old rhizomes > three-year-old rhizomes > two-year-old rhizomes > roots, with leaf concentrations approximately 10 times more than those in rhizomes and autumn (November) identified as the optimal harvest period (Lin et al., 2019) (Figure 3B). Although the Chinese Pharmacopoeia uses bergenin as the quality marker, which predominantly accumulates in rhizomes, the utilization of leaves for gallic acid and arbutin extraction also shows promising potential.

## 4 Chemical metabolite and quality control

### 4.1 Chemical metabolite

#### 4.1.1 Phenolics

Phenolics are the active metabolites of *B. purpurascens* and occur throughout the plant (Min, 2007; Chen et al., 1987; Yan et al., 2024). Major phenolics include bergenin, gallic acid and its derivatives, arbutin, and catechin (Table 5; Figure 5). Bergenin, a 4-O-methyl gallic acid C-glycoside, is the primary active metabolite isolated from *B. purpurascens* and is listed in the Pharmacopoeia of the People’s Republic of China as an antitussive and expectorant agent for the clinical treatment of respiratory diseases such as chronic bronchitis (Liu et al., 2023; Yan et al., 2024). A study by Professor Yang Lin’s team (Yan et al., 2024), published in Nature Communications, first elucidated bergenin’s complete biosynthetic pathway. By reconstructing and optimizing its *de novo* biosynthesis in *Escherichia coli*, large-scale production was achieved, potentially addressing limitations in clinical supply. Subcellular localization studies by Taneyama and Yoshida found that bergenin is primarily distributed in vacuoles, whereas gallic acid is present in both vacuoles and chloroplasts in comparable amounts. Tracer experiments further suggest that gallic acid serves as a precursor of bergenin (Taneyama and Yoshida, 1979). Related studies have confirmed that bergenin biosynthesis proceeds from gallic acid via sequential catalysis by 2-C-glycosyltransferase and 4-O-methyltransferase, forming 4-O-methyl gallic acid 2-C-β-D-glucoside, which is then esterified to produce bergenin (Yan et al., 2024). Detection across five plant parts showed that gallic acid content is highest in leaves and lowest in fibrous roots, indicating that leaves are the primary site of gallic acid synthesis and accumulation in *B. purpurascens* (Liu et al., 2015). Metabolomic analyses of *B. purpurascens* identified 351 differentially accumulated secondary metabolites across the leaves and stems, with leaves having the highest accumulation of phenolics (Zhu et al., 2024). Integrated transcriptomic–metabolomic analyses further identified five UDP-glycosyltransferases and four O-methyltransferases as candidate enzymes involved in the biosynthetic pathway from gallic acid to bergenin (Zhu et al., 2024). Collectively, these findings indicate that phenolics underlie the diverse biological activities of *B. purpurascens*.

**TABLE 5:** Classification of phenolics in *Bergenia purpurascens*.

No.	Classification	Quantity	Core structure	Name	Plant source	CAS no.	PubChem CID	References
1	Coumarins	8	Isocoumarin glycoside	Bergenin	*B. purpurascens* *B. scopulosa* *B. crassifolia*	477-90-7	66065	(Shi, 2013; Lv and Wang, 2003; Cai, 2023; Katanić Stanković et al., 2022; Salminen et al., 2014)
2	Phenolic glycosides	5	Hydroquinone-O-glycoside	Arbutin	*B. purpurascens* *B. scopulosa* *B. crassifolia*	497-76-7	440936	(Shi, 2013; Lv and Wang, 2003; Cai, 2023; Katanić Stanković et al., 2022)
3	Phenolic acids	12	Benzoic acids	Gallic acid	*B. purpurascens* *B. crassifolia* *B. scopulosa*	149-91-7	370	(Shi, 2013; Lv and Wang, 2003; Cai, 2023; Katanić Stanković et al., 2022; Salminen et al., 2014)
4	—	—	—	Protocatechuic acid	*B. purpurascens* *B. crassifolia*	99-50-3	72	(Shi, 2013; Cai, 2023; Katanić Stanković et al., 2022; Salminen et al., 2014)
5	—	—	—	Vanillic acid	*B. purpurascens* *B. crassifolia*	121-34-6	8468	(Shi, 2013; Cai, 2023; Katanić Stanković et al., 2022; Salminen et al., 2014)
6	—	—	—	Syringic acid	*B. purpurascens* *B. crassifolia*	530-57-4	10742	(Shi, 2013; Cai, 2023; Katanić Stanković et al., 2022; Taneyama and Yoshida, 1979)
7	—	—	—	Catechol	*B. purpurascens*	120-80-9	289	(Shi, 2013; Cai, 2023; Salminen et al., 2014)
8	—	—	Cinnamic acids	Caffeic acid	*B. purpurascens* *B. crassifolia*	331-39-5	689043	(Shi, 2013; Cai, 2023; Katanić Stanković et al., 2022; Salminen et al., 2014)
9	—	—	—	Ferulic acid	*B.purpurascens* *B.crassifolia*	1135-24-6	445858	(Shi, 2013; Cai, 2023; Katanić Stanković et al., 2022; Salminen et al., 2014)
10	—	—	—	p-Coumaric acid	*B. crassifolia*	501-98-4	637542	(Shi, 2013; Katanić Stanković et al., 2022; Koul et al., 2020)
11	—	—	—	Sinapic acid	*B. crassifolia*	530-59-6	637775	(Shi, 2013; Katanić Stanković et al., 2022; Koul et al., 2020)
12	—	—	Quinic acids	Chlorogenic acid	*B.purpurascens* *B. crassifolia*	327-97-9	1794427	(Shi, 2013; Cai, 2023; Katanić Stanković et al., 2022; Salminen et al., 2014)
13	—	—	—	Quinic acid	*B. crassifolia*	77-95-2	6508	(Shi, 2013; Katanić Stanković et al., 2022; Koul et al., 2020)
14	Flavonoids	9	Flavan-3-ols	(+)-Catechin	*B.purpurascens* *B. scopulosa* *B. crassifolia*	154-23-4	9064	(Shi, 2013; Lv and Wang, 2003; Cai, 2023; Katanić Stanković et al., 2022; Salminen et al., 2014)
15	—	—	—	Epicatechin	*B. crassifolia* *B. scopulosa*	490-46-0	72276	(Shi, 2013; Lv and Wang, 2003; Cai, 2023; Katanić Stanković et al., 2022)
16	—	—	—	Epicatechin gallate	*B. crassifolia*	1257-08-5	107905	(Shi, 2013; Lv and Wang, 2003; Katanić Stanković et al., 2022; Koul et al., 2020)
17	—	—	Flavonols	Kaempferol	*B. crassifolia*	520-18-3	5280863	(Shi, 2013; Lv and Wang, 2003; Katanić Stanković et al., 2022; Koul et al., 2020)
18	—	—	—	Quercetin	*B. crassifolia*	117-39-5	5280343	(Shi, 2013; Li et al., 2011; Lv and Wang, 2003; Katanić Stanković et al., 2022)
19	—	—	—	Quercitrin	*B. crassifolia*	522-12-3	5280459	(Shi, 2013; Lv and Wang, 2003; Katanić Stanković et al., 2022; Koul et al., 2020)
20	—	—	Dihydroflavonoids	Naringenin	*B. purpurascens*	480-41-1	932	(Shi, 2013; Cai, 2023; Salminen et al., 2014)
21	Tannins	11	Ellagitannins	Ellagic acid	*B. crassifolia, B. purpurascens*	476-66-4	5281855	(Shi, 2013; Lv and Wang, 2003; Katanić Stanković et al., 2022; Koul et al., 2020)

**FIGURE 5:**
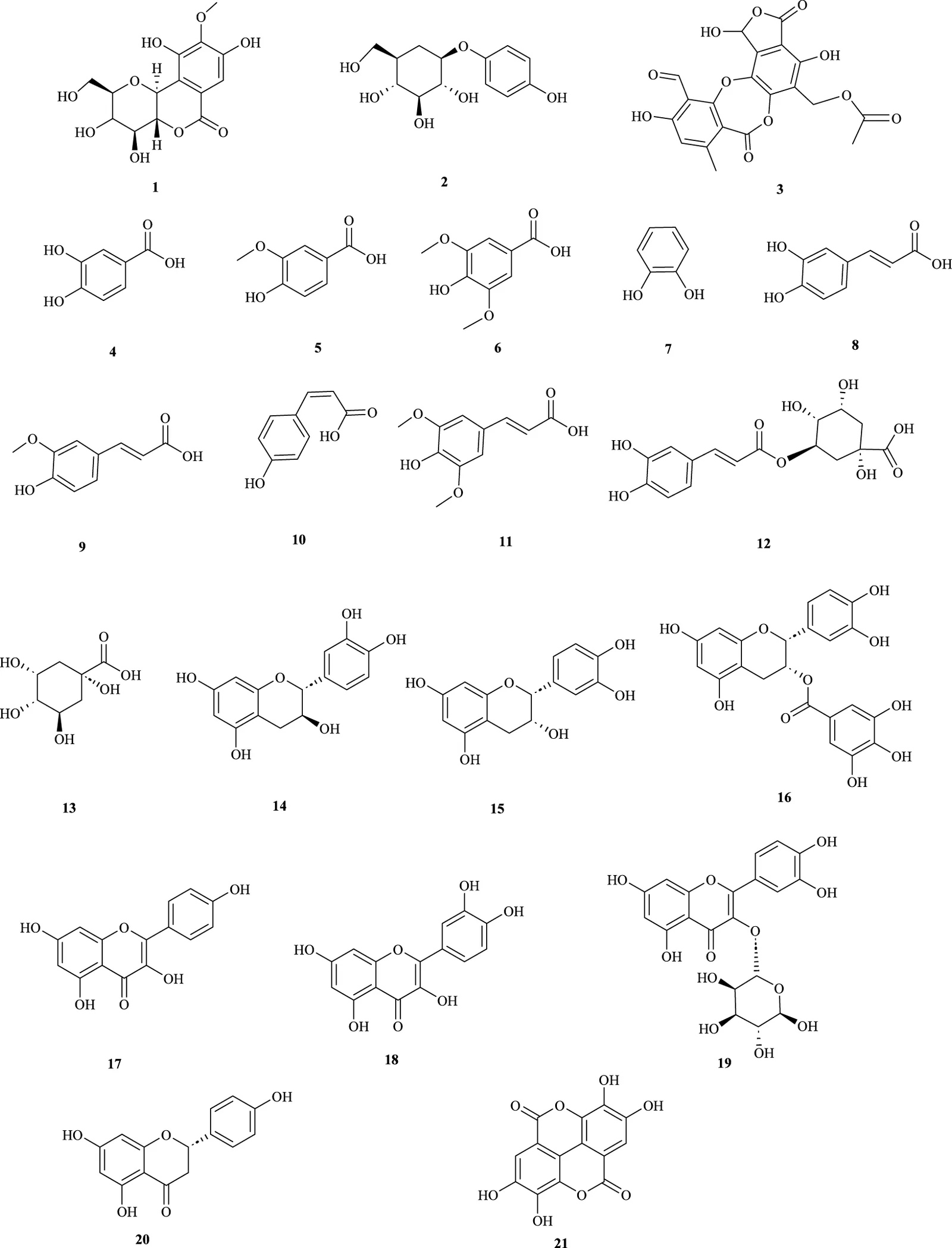
Chemical structures of phenolics in *Bergenia purpurascens*.

#### 4.1.2 Sulfoxides

Studies have reported that substantial levels of diethyl disulfoxide, a sulfur-containing metabolite, can be isolated from the whole plant of *B. purpurascens* (Xia et al., 2017; Shi, 2013). This metabolite is rare in plants and has previously been found only in durian (*Durio zibethinus* Murr.) of the Bombacaceae family (Nf and Velluz, 1996).

#### 4.1.3 Polysaccharides

Polysaccharides are important active metabolites of *B. purpurascens* in addition to phenolics and are widely distributed in the rhizomes of *Bergenia* species (Xia et al., 2017). The harvest period also significantly affects their content (Zhu et al., 2024). For example, the polysaccharide content in the rhizomes of *B. emeiensis* is higher than that in other species (Chen et al., 2009), indicating interspecific and tissue-level variation. The optimal extraction method is alcohol precipitation, and the content is commonly determined using the phenol-sulfuric (Lin et al., 2005) and anthrone-sulfuric acid methods (Feng et al., 2023). Current studies on *Bergenia* polysaccharides mainly focus on extraction optimization and content determination, with limited investigation of detailed chemical structures. Methyl-α-D-fructofuranoside (BIN-5), isolated from *B. crassifolia*, is the first sugar metabolite isolated from this genus and provides a reference for structural studies of *Bergenia* polysaccharides (Liu, 2016). Therefore, in-depth research on their chemical structures and structure–activity relationships is required.

#### 4.1.4 Sterols

β-Sitosterol has been shown to exert anti-inflammatory effects by binding to and inhibiting the COX-2 active site (Cai, 2023). Sterol metabolites identified in *B. purpurascens* (Table 6; Figure 6) mainly include β-sitosterol and daucosterol (β-sitosterol-3-O-glucoside). A systematic study of the underground parts of *B. scopulosa* isolated and identified these metabolites for the first time (Lv and Wang, 2003). Subsequent studies using various chromatographic methods, including macroporous resin column chromatography, confirmed their presence in methanol extracts (Wei, 2012). Similarly, an investigation of the active metabolites of the Xinjiang specialty plant *B. crassifolia* identified 12 metabolites from ethyl acetate and *n*-butanol fractions, including β-sitosterol and daucosterol (Liu, 2016). Additionally, stigmasterol was isolated from the ethyl acetate fraction of *B. scopulosa* (Liu et al., 2012). These results indicate that sterols are important lipophilic metabolites of *B. purpurascens* and may contribute to its anti-inflammatory activity.

**TABLE 6:** Classification of sterol metabolites in *Bergenia purpurascens*.

No.	Name	Molecular formula	Plant source	Extraction part	CAS no.	PubChem CID	References
1	β-Sitosterol	C_29_H_50_O	*B. scopulosa*	Rhizome	83-46-5	222284	(Lv and Wang, 2003; Liu, 2016; Wei, 2012; Cui, 2011; Hasan, 2021; Chauhan and Sharma, 2012)
			*B. crassifolia*	Rhizome	—	—	—
			*B. ligulata*	Rhizome	—	—	—
2	Daucosterol	C_35_H_60_O_6_	*B. scopulosa*	Rhizome	474-58-8	5742590	(Lv and Wang, 2003; Liu, 2016; Wei, 2012; Cui, 2011)
			*B. crassifolia*	Rhizome	—	—	—
3	Stigmasterol	C_29_H_48_O	*B. scopulosa*	Rhizome	83-48-7	5280794	(Liu et al., 2012; Cui, 2011; Chauhan and Sharma, 2012)
		C_29_H_48_O	*B. ligulata*	Rhizome	—	—	—
4	Campesterol	C_28_H_48_O	*B. crassifolia*	Rhizome	474-62-4	173183	(Koul et al., 2020)

**FIGURE 6:**
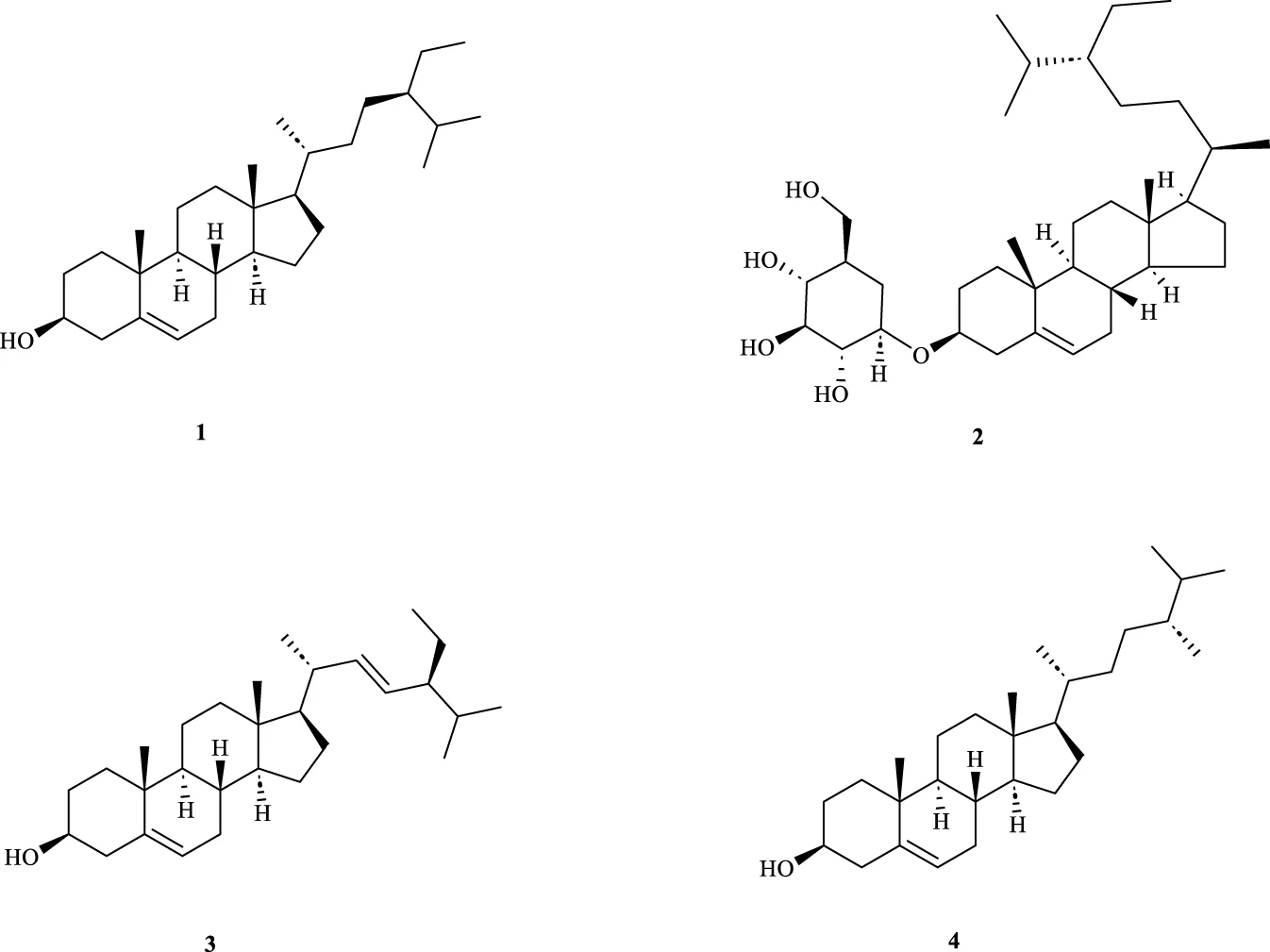
Chemical structures of sterol metabolites in *Bergenia purpurascens*.

### 4.2 Quality control

Bergenin and arbutin are the two important polyphenolics of *B. purpurascens* (Chen et al., 1987; Okuda, 1987). Therefore, the Chinese Pharmacopoeia (2025 Edition, Part I) specifies thin-layer chromatography identification for both metabolites; bergenin is also listed as an antitussive and expectorant drug in the Chinese Pharmacopoeia (2025 Edition, Part II) (National Pharmacopoeia Committee, 2025). Multiple studies indicate the bergenin content in *B. purpurascens* is not significantly affected by regional differences (Shu and Zhang, 1991; Jiang et al., 2010; Ji, 2005; Wang et al., 2010), supporting its use as a marker metabolite for quality control of *B. purpurascens* medicinal material. Furthermore, bergenin, catechin, arbutin, and gallic acid are the main phenolics in *B. scopulosa*. An high-performance liquid chromatography (HPLC) method enabling simultaneous determination of these four metabolites has been established, providing a technical basis for improved quality control of *B. purpurascens* (Min, 2007).

## 5 Pharmacological effects

Recent studies have indicated that the extracts of *B. purpurascens* and its primary metabolite bergenin exhibit various pharmacological activities, including expectorant, antitussive, antibacterial, anti-inflammatory, anti-peptic ulcer, hepatoprotective, astringent and hemostatic, antitumor, neuroprotective, antidiabetic, and hypolipidemic effects (Liu et al., 2023; Pandey et al., 2025) (Figure 7).

**FIGURE 7:**
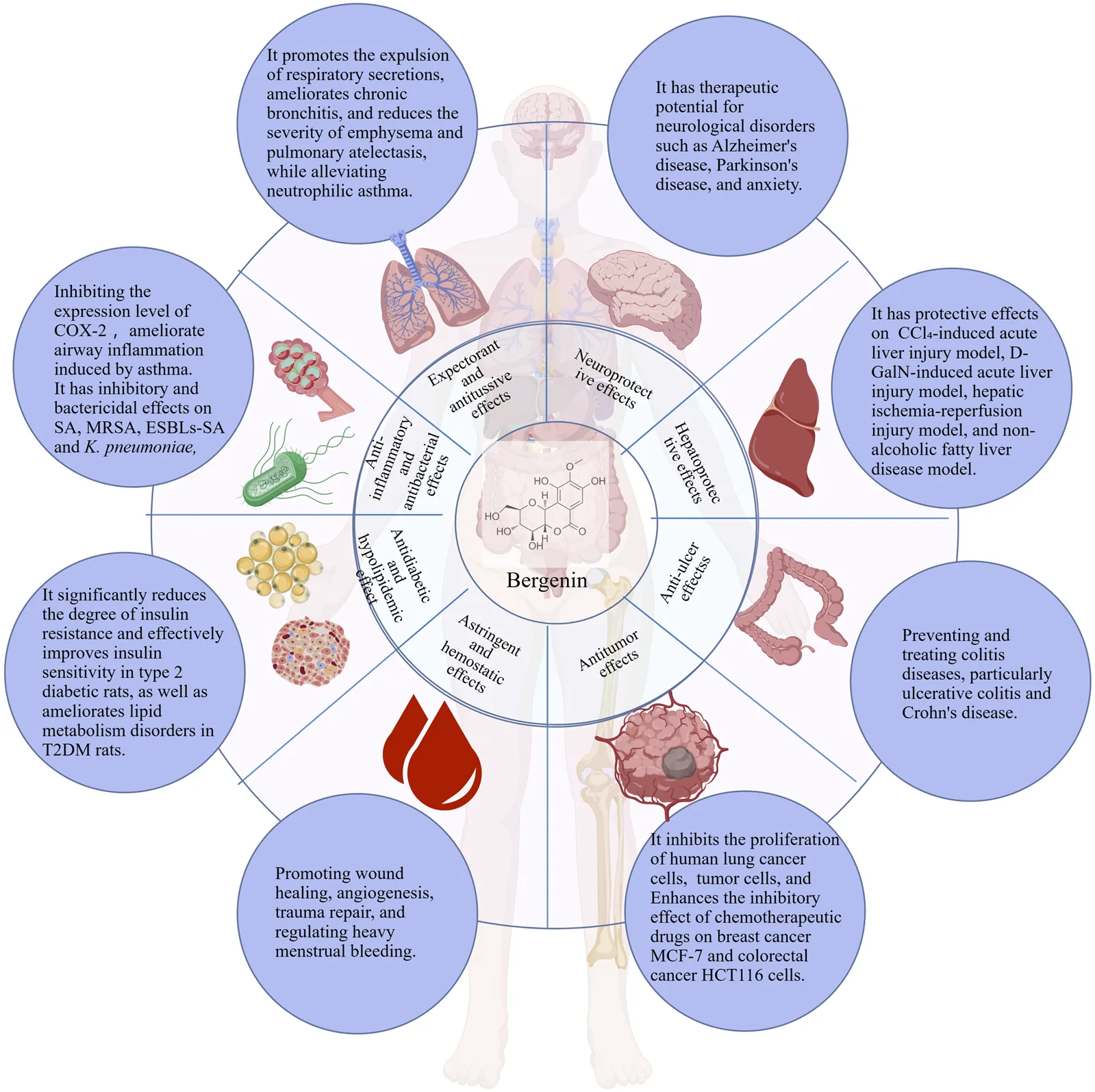
Pharmacological effects of bergenin.

### 5.1 Expectorant and antitussive effects

Pharmacological studies have shown that bergenin, the main active metabolite of *B. purpurascens*, exerts significant expectorant and antitussive effects and is included in the Pharmacopoeia of the People’s Republic of China as an antitussive and expectorant drug (National Pharmacopoeia Committee, 2025). Its action involves a central antitussive mechanism. Animal experimental studies have shown that bergenin exhibits significant antitussive activity at a dose of 100 mg/kg, with efficacy of approximately 1/7 to 1/4 that of codeine phosphate, and no drug resistance develops after continuous administration for >20 days (Zhou et al., 2020). It also significantly increases tracheal phenol red excretion in mice, thereby promoting respiratory secretion clearance (Zhang et al., 2026), and alleviates pulmonary emphysema and atelectasis in rat models of chronic bronchitis. Recent studies have further elucidated its molecular mechanisms in treating respiratory diseases. As a SIRT1 activator, bergenin deacetylates NF-κB and inhibits its nuclear translocation in macrophages, thereby reducing interleukin-1β (IL-1β), IL-6, and MMP-9 production and improving asthma-induced airway inflammation and remodeling (Huang et al., 2022). Furthermore, as a natural peroxisome proliferator-activated receptor γ (PPAR-γ) agonist, it regulates the CDK1-APC/C-Cdh1 signaling pathway, inhibits GLS1-dependent glutaminolysis, and reduces the proportion of CD4^+^ IL-17A^+^ T cells and RORγt expression, ultimately alleviating neutrophilic asthma (Yang et al., 2022). These findings indicate that bergenin exerts antitussive and expectorant effects through multiple mechanisms, including central antitussive action, enhanced secretion clearance, anti-inflammatory repair, and immune regulation (Figure 8).

**FIGURE 8:**
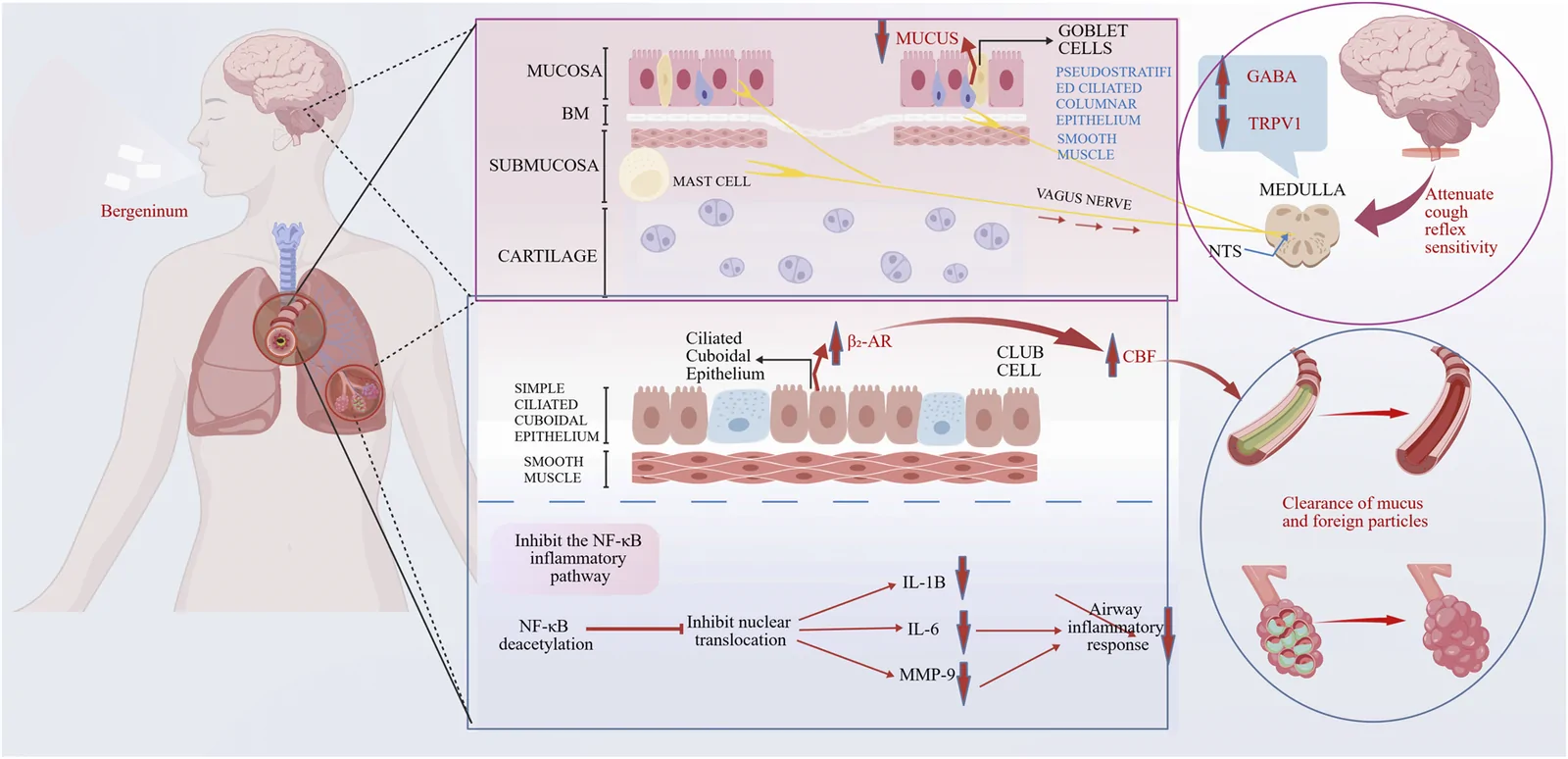
Mechanism of antitussive and expectorant action of bergenin.

### 5.2 Antibacterial effects

Extracts of *B. purpurascens* exhibit inhibitory and bactericidal activity against *Staphylococcus aureus* (SA), methicillin-resistant *S. aureus*, and β-lactamase-producing *S. aureus* (ESBLs-SA) (Shi et al., 2014). Bergenin, administered at low (1.5 μg/mL), medium (3 μg/mL), and high (6 μg/mL) doses, alleviates lung injury and reduces pulmonary bacterial load by improving the decreased cell viability, oxidative damage, and inflammatory responses induced by *Klebsiella pneumoniae* infection and by inhibiting the phosphorylation of ERK, JNK, p38, IκB, and p65 (Hao et al., 2020). Bergenin lactone also shows antifungal activity against seven common pathogenic fungi, including *Penicillium citrinum*, *Penicillium digitatum*, and *Colletotrichum gloeosporioides*, with the strongest effect against *P. citrinum*; at 20 μg/mL, it induces significant morphological changes in mycelia (Zuo et al., 2012). The methanol extracts of *B. purpurascens* exert antibacterial effects against food-spoilage bacteria such as *E. coli*, *Staphylococcus epidermidis*, and *Shigella* spp., with inhibition increasing with concentration, supporting its potential application as a natural preservative (Cheng et al., 2016).

### 5.3 Anti-inflammatory effects

The ethyl acetate extract of *B. purpurascens* significantly inhibits xylene-induced ear edema in mice, confirming its anti-inflammatory activity (Shi et al., 2014). *Bergenia* extract (100, 200, and 400 mg/kg) significantly inhibited xylene-induced ear edema in mice and acetic acid–induced increase in abdominal capillary permeability, and this inhibitory effect was enhanced with increasing doses. Screening using a COX-2 fluorescence inhibitor assay demonstrated that the ethyl acetate fraction exerted the strongest inhibitory effect. From this fraction, a total of 38 metabolites, including β-sitosterol, caryophyllene, 11-O-(4-O-hydroxybenzoyl)-bergenin, chlorogenic acid, and catechin, were identified, with chlorogenic acid exhibiting the strongest inhibitory activity (Cai, 2023). These findings suggest that phenolic acids and flavonoids constitute the primary material basis for the antibacterial and anti-inflammatory effects of *Bergenia* species. A network pharmacology study predicted that the key therapeutic targets of *Bergenia* in osteoarthritis include AKT1, MAPK1, and MAPK3, involving signaling pathways associated with apoptosis, estrogen, and MAPK signaling. The study further suggested that bergenin, 11-O-galloylbergenin, arbutin, and catechin-3-O-gallate exert synergistic therapeutic effects against osteoarthritis (Qu et al., 2020).

### 5.4 Anti-peptic ulcer effects

Bergenin has recently been confirmed to exert significant anti-gastrointestinal ulcer effects (Salimo et al., 2023). The intravenous administration of bergenin at a dose of 30 mg/kg has been reported to effectively prevent stress-induced gastric ulcers in rats (Abe et al., 1980). Patent data indicate its efficacy in preventing and treating colitis, particularly ulcerative colitis and Crohn’s disease. Mechanistic studies have also shown that bergenin alleviates DSS-induced ulcerative colitis in mice by reducing *Bacteroides vulgatus*-mediated increases in branched-chain amino acids, inhibiting TLR4, and regulating NF-κB and mTOR phosphorylation (Huang et al., 2024). Further studies demonstrate that bergenin upregulates miR-124-3p expression, thereby downregulating carnitine palmitoyltransferase 1-mediated fatty acid oxidation, reducing H3K27 acetylation in the RORγt promoter region of γδT17 cells, and inhibiting γδT17 activation and IL-17 production, thus alleviating ulcerative colitis (Guo et al., 2025). These findings provide a scientific basis for its application in gastrointestinal ulcers and inflammatory bowel disease.

### 5.5 Hepatoprotective effects

*Bergenia purpurascens* and bergenin exert significant hepatoprotective effects across various liver injury models. In CCl_4_-induced acute liver injury in rats, bergenin significantly reduces serum levels of alanine aminotransferase, aspartate aminotransferase, and malondialdehyde (MDA), as well as hepatic levels of tumor necrosis factor-α (TNF-α), IL-6, and IL-1β, increasing serum levels of albumin, superoxide dismutase (SOD), and glutathione peroxidase (GSH-Px) and inhibiting IL-6/JAK2/STAT3 pathway-related protein expression (Lan et al., 2021). Similar effects have been observed in D-galactosamine-induced acute liver injury, further validating its hepatoprotective activity (X et al., 2022). In a hepatic ischemia-reperfusion injury model, bergenin exerted hepatoprotective effects by scavenging reactive oxygen species (ROS), modulating inflammatory cytokines, and regulating apoptosis- and autophagy-related genes via the PPAR-γ signaling pathway. It reduces ROS production, downregulates TNF-α, IL-6, and IL-1β, inhibits apoptosis and autophagy, and dose-dependently increases PPAR-γ-related gene expression while reducing the phosphorylation of P38 MAPK, NF-κB p65, and JAK2/STAT1-related proteins (Xiang et al., 2020). In nonalcoholic fatty liver disease models, bergenin significantly improves liver dysfunction in high-fat diet-induced C57 mice by regulating the SIRT1/NF-κB and AMPK/Nrf2 pathways. It significantly downregulates p-NF-κB p65, TNF-α, IL-6, IL-1β, SREBP-1, and FAS expression, while upregulating SIRT1, AMPK, Nrf2, and HO-1 expression, thereby reducing inflammation and oxidative stress, improving lipid metabolism, and exerting hepatoprotective effects (Li and an, 2022).

### 5.6 Astringent and hemostatic effects

The astringent and hemostatic effects of *B. purpurascens* are clearly documented in both traditional medicine and modern pharmacological studies. In traditional medicine, it is described as sweet, slightly astringent, and cool in nature, with effects of cooling blood and hemostasis, and is used for various traumatic bleeding. Evidence indicates fresh leaves of *B. purpurascens* applied to fresh wounds exert superior hemostatic effects compared with dried materials (Tian and Hu, 2007). Modern pharmacological studies further show that the rhizome extract of *B. ciliata* significantly accelerates wound healing (Imran et al., 2023). The chemical analyses of *B. pacumbis* rhizomes identified bergenin and its derivative 11-O-galloylbergenin as the active metabolites that promote wound healing, angiogenesis, and regulation of excessive menstrual bleeding (Munankarmi et al., 2026). Additionally, animal experiments have confirmed that the rhizome extracts of *B. purpurascens* significantly accelerate wound closure, promote epithelial regeneration, and enhance collagen synthesis, providing a scientific basis for its traditional use in hemostasis and tissue repair (Faiz et al., 2023).

### 5.7 Antitumor effects

Bergenin effectively inhibits the proliferation of various tumor cells through multiple pathways and targets. The extract of *B. emeiensis* significantly inhibits the proliferation of human lung cancer A549 cells *in vitro* in a concentration-dependent manner. It also exhibits significant antitumor activity in a mouse hepatocarcinoma (S180) model, achieving a maximum tumor inhibition rate of 52.45% at an intragastric dose of 400 mg/kg (Liu et al., 2013). Bergenin exerts time- and dose-dependent inhibitory effects on the proliferation of the non-small cell lung cancer cell line HCC827. As the concentrations increase (7.5, 15, and 30 μmol L^−1^), the proliferation inhibition rate also increases. This effect may be associated with the inhibition of the NF-κB signaling pathway, as bergenin significantly reduces NF-κB p65 expression while significantly increasing apoptosis (Zhang and Tian, 2022). In malignant gliomas, bergenin specifically inhibits the proliferation, colony formation, migration, and invasion of U251 and U87 cells while inducing apoptosis. These effects are mediated through the downregulation of the PI3K–AKT–mTOR pathway phosphorylation. Moreover, it shows a dose-dependent activity (0, 0.5, 1, 2, 4, and 8 μM) and strong selectivity, showing no significant cytotoxicity against normal human glial HEB cells (Tian et al., 2023). Recent studies further confirm that bergenin inhibits tumor proliferation by stimulating intracellular ROS production, inducing DNA damage, and causing cell cycle arrest at G1/G2 phases (Pandey et al., 2025). Furthermore, when combined with cisplatin or 5-fluorouracil, bergenin significantly enhances the inhibitory effects of these chemotherapeutic agents on breast cancer MCF-7 and colorectal cancer HCT116 cells (Kumar et al., 2025), suggesting potential dose reduction in chemotherapy.

### 5.8 Neuroprotective effects

Bergenin has neuroprotective potential. Both *in vitro* and *in vivo* studies show that it modulates the activity of key enzymes and proteins, including reelin, GSK-3β, Lingo-1, Aβ 1–42, P-tau, SOD, GPx, BDNF, and VEGF, and regulates cytokines such as IL-6, IL-1β, TNF-α, and TGF-β. These effects involve multiple signaling pathways, including PI3K/Akt, NF-κB, PKC, Nrf2, and Sirt1/FOXO3a. Preclinical studies indicate therapeutic potential in neurological disorders, including Alzheimer’s disease, Parkinson’s disease, anxiety, depression, addiction, epilepsy, insomnia, and stroke (Rezaiezadeh et al., 2024). In a spinal cord injury model, bergenin significantly promoted histopathological repair and motor function recovery in rats, reduced lesion cavity area, increased myelin preservation, and promoted the survival of the anterior horn motor neurons. It also reduced H_2_O_2_ and MDA levels in spinal cord tissue, enhanced SOD and GSH-Px activity, downregulated IL-1β, IL-6, and TNF-α expression, and promoted M1-to-M2 macrophage polarization via the activation of the PPARγ signaling pathway (Sheng, 2022). In a cellular oxidative stress model, bergenin protected SH-SY5Y human neuroblastoma cells from H_2_O_2_-induced injury by significantly improving cellular morphology, reducing lactate dehydrogenase (LDH) release and ROS production, decreasing MDA levels, and inhibiting mitochondrial membrane potential loss and apoptosis. Mechanistically, this involves the activation of the PI3K/Akt signaling pathway, increasing the levels of antioxidant proteins SOD1 and SOD2, increasing the Bcl-2/Bax ratio, and reducing cleaved caspase-3 expression (Hu, 2021). In a cerebral ischemia-reperfusion injury model, bergenin scavenged free radicals in mouse brain tissue, inhibited lipid peroxidation, and protected brain tissue (Pu, 2006). In addition, bergenin can cross the blood-brain barrier, with an apparent permeability coefficient of (1.07 ± 0.27) × 10^–6^ cm s^–1^ and an efflux ratio of 4.32 in the MDCK-MDR1 model. Co-administration with the P-glycoprotein inhibitor verapamil significantly increases its permeability, indicating moderate blood-brain barrier permeability mediated by both P-glycoprotein transport and passive diffusion (Wu et al., 2022).

### 5.9 Other effects

Studies have shown that *B. purpurascens* also exerts antidiabetic and hypolipidemic effects. Patent-reported animal studies indicate that bergenin significantly reduces the insulin resistance and effectively improves insulin sensitivity in type 2 diabetic rats. It also significantly reduces serum triglyceride and total cholesterol (TC) levels, significantly improving lipid metabolism disorders in type 2 diabetic rats (Jin and Sun, 2012; Quan et al., 2023). In hyperlipidemic mice, bergenin significantly reduces serum TC and low-density lipoprotein cholesterol levels, while significantly increasing high-density lipoprotein cholesterol levels. It also significantly increases SOD and GSH-Px activities in serum and liver tissues to near-normal levels, showing stronger effects than vitamin E at the same dose (250 mg/kg) (Yang, 2004). Using a UVB-induced mouse skin damage model, the ameliorative effect of *B. purpurascens* extract (BPE) on UV-induced injury was evaluated. An α-MSH-induced melanogenesis model in B16F10 mouse melanoma cells was used to assess the *in vitro* activities of BPE and its primary active component, bergenin, and an α-MSH-stimulated zebrafish melanogenesis model was used to verify the antipigmentary activity of bergenin. BPE significantly inhibited UVB-induced skin damage in mice and restored skin barrier function *in vivo*. Both BPE and bergenin significantly inhibited α-MSH-induced melanin overproduction in B16F10 cells, with bergenin at 10 μg/mL exhibiting superior efficacy compared to the well-known whitening agent α-arbutin. For the first time, BPE and bergenin were found to exert antimelanogenic and skin-whitening effects by targeting TYR and TRP-2 proteins, providing a scientific basis for their traditional applications. Therefore, bergenin was confirmed to possess potent antimelanogenic and skin-whitening properties through the targeting of tyrosinase and tyrosinase-related protein-2 (Liu et al., 2024). Previous studies have also confirmed that the ethanol extract of *B. purpurascens* exhibits potent antioxidant activity and inhibits mushroom tyrosinase activity more effectively than the positive control arbutin. Furthermore, the ethanol extract suppresses melanin production through a dual mechanism: direct inhibition of tyrosinase activity and downregulation of melanogenesis-related genes, including *Trp-1*, *Trp-2*, and tyrosinase, by suppressing the MITF-mediated downstream signaling pathway (Seo et al., 2024). Another study found that bergenin is a potential xanthine oxidase inhibitor; by inhibiting the activity of this enzyme, it may reduce uric acid production and thus have therapeutic potential for hyperuricemia and gout (Liang et al., 2023).

## 6 Toxicology

Within the conventional usage range, *B. purpurascens* is considered nontoxic or of low toxicity, and its main active metabolite, bergenin, exerts hepatoprotective effects (Xu et al., 2022). However, it should not be co-administered with drugs metabolized via testosterone, chlorzoxazone, or diclofenac (Liu et al., 2023). Early genotoxicological studies indicate that *B. purpurascens* extracts do not increase micronucleus formation, chromosomal aberrations, or sister chromatid exchange frequency at appropriate doses, although toxicity occurs at excessively high doses (Cai, 1991). Notably, Qu et al., through network pharmacology analysis, predicted that bergenin exhibits potential binding activity with CYP2C9 and CYP2E1, suggesting a potential inhibitory effect on these enzymes. However, direct *in vitro* CYP inhibition assays, such as those using human liver microsomal systems, are currently lacking to validate this prediction. Furthermore, bergenin exhibits low oral bioavailability (OB), as indicated by predicted OB values, which is consistent with previous findings describing its poor water solubility and susceptibilty to intestinal efflux. Although low OB may reduce the risk of systemic toxicity, its potential effects on the gut microbiota and intestinal metabolic enzymes warrants attention (Qu et al., 2020). Liu et al. identified cuscutin as the key component responsible for the whitening activity of *B. purpurascens* (Liu et al., 2024). Seo et al. demonstrated that the ethanol extract inhibits tyrosinase through multiple mechanisms. Collectively, these findings provide an important basis for further safety assessments of *B. purpurascens* and its major constituents, including bergenin and cuscutin. However, toxicological research—particularly regarding CYP enzyme inhibition, genotoxicity, and long-term toxicity—remains substantially limited compared with pharmacological research (Seo et al., 2024). Despite its broad pharmacological activities, comprehensive toxicological evaluations remain limited, and further studies are required to support its safe clinical application.

## 7 Modern product applications

*Bergenia purpurascens* has long been used as a traditional Chinese medicinal material, recorded in pharmacopoeias such as Emei Medicinal Plants and Sichuan Chinese Materia Medica. Its leaves are edible and used to clear heat, reduce inflammation, and relieve pain; tender leaves can be stir-fried and prepared as tea. Fresh or dried plants are also used in making soups and may alleviate asthma and dry cough (Lv et al., 2017). The traditional Chinese medicine preparation “Compound Bergenin Tablet,” a formulation with bergenin as the main active component, has been successfully launched in China and showed effective antitussive outcomes in 30 patients with post-cold cough (Zhou and Wang, 2014). It is currently used clinically to treat chronic bronchitis. *Bergenia purpurascens* is also used in the development of compound preparations for treating peptic ulcers. A 2014 patent (CN104042890A) describes a pharmaceutical composition for treating peptic ulcers comprising 14 medicinal materials, including *Angelica dahurica*, *Codonopsis pilosula*, and *B. purpurascens,* characterized by low cost, good efficacy, minimal toxic side effects, and low tolerance potential, providing a new option for clinical medication. Beyond pharmacological uses, *B. purpurascens* has applications in cosmetics and landscaping. Traditional Tibetan sunscreen formulations use it as a main metabolite, and Tibet University has developed and industrialized a sunscreen product with significant efficacy in ultraviolet absorption (Ouzhu et al., 2011). For *B. scopulosa*, systematic evaluation of its adaptability, ornamental characteristics, and landscape application value as a garden ornamental plant, based on its appearance, flowering period, ecological benefits, etc., confirms its suitability for urban greening (Cai, 2025). Overall, *B. purpurascens* shows promising development potential, including future applications in anti-aging and anti-inflammatory skincare products, as well as external hemostatic formulations such as ointments or powders (Figure 9).

**FIGURE 9:**
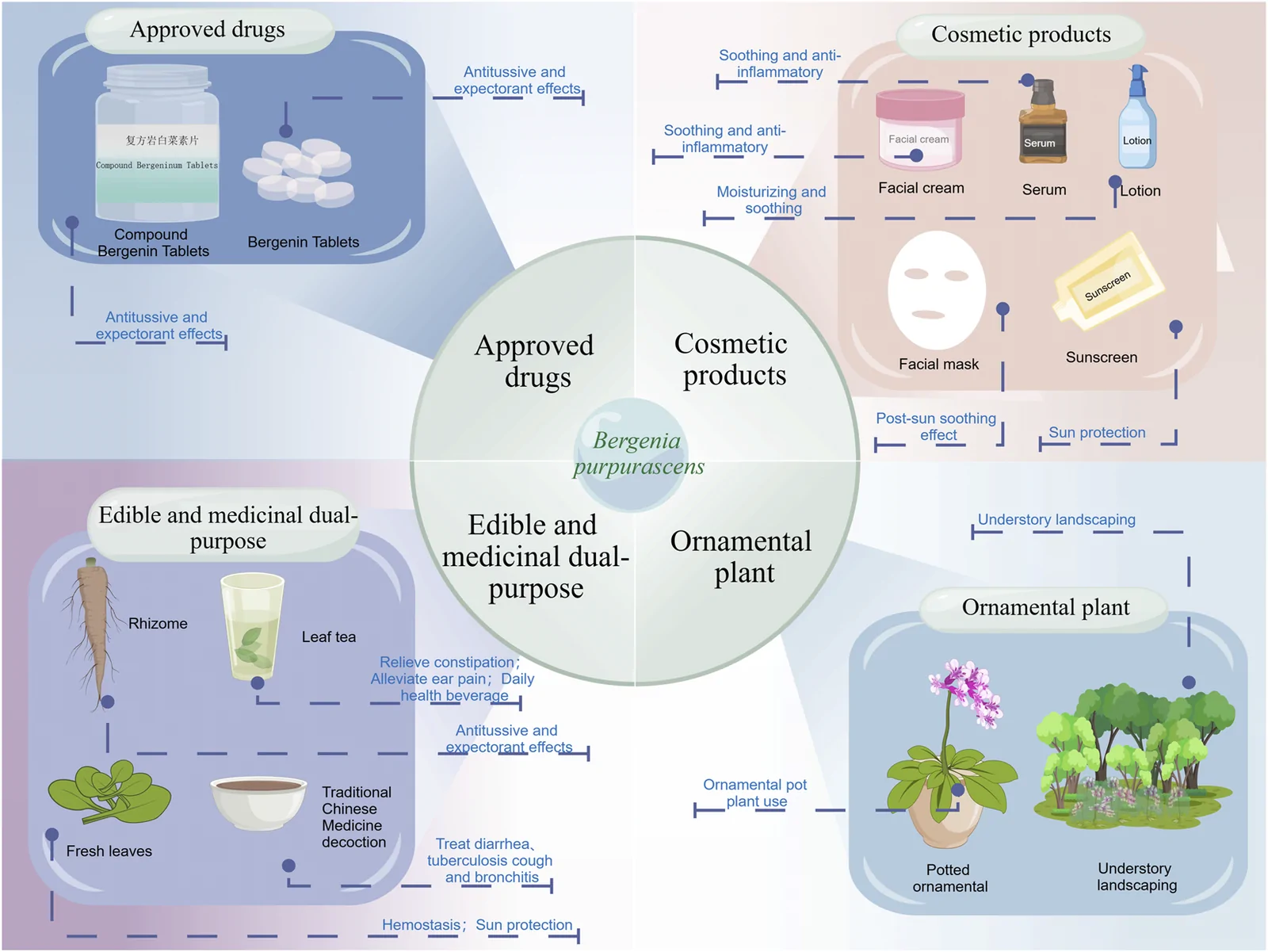
Modern product applications of *Bergenia purpurascens*.

## 8 Discussion

*Bergenia purpurascens* is a perennial herb with a long history of medicinal use in southwestern China and the Himalayan region. Its dried rhizome is listed in the Pharmacopoeia of the People’s Republic of China for the treatment of respiratory diseases, including chronic bronchitis. This review systematically summarizes the research progress on its botanical characteristics, resource distribution, chemical constituents, pharmacological activities, toxicology, and modern applications.

The active constituents of *B. purpurascens* are phenolics, including bergenin, gallic acid, and arbutin, which exhibit expectorant, antitussive, anti-inflammatory, hepatoprotective, antitumor, and neuroprotective activities. Recent advances in synthetic biology have elucidated the complete biosynthetic pathway of bergenin, enabling its large-scale production in engineered *E. coli* and potentially addressing clinical supply limitations. In addition, polysaccharides and sterols have been identified as active components, although their chemical structures and seasonal variations require further investigation.

Currently, the medicinal materials of *B. purpurascens* are sourced primarily from wild populations. Habitat degradation in high-altitude regions and over-harvesting may lead to a decline in the genetic diversity of some populations, particularly within the China–Himalaya biodiversity hotspot. Research on artificial cultivation techniques, including tissue culture and Good Agricultural Practice (GAP)-standardized cultivation systems, remains at a nascent stage and is far from large-scale commercial promotion. Existing quality evaluation standards almost exclusively rely on bergenin content, as specified in the Chinese Pharmacopoeia. However, significant chemotypic variation in other active phenolics, including arbutin and gallic acid, exist across different producing areas, harvest periods, and plant parts (e.g., rhizomes vs. leaf). Consequently, a quality control system based solely on a single indicator may not adequately reflect overall efficacy.

Modern pharmacological studies have validated the traditional applications of *B. purpurascens* at the molecular level, revealing mechanisms involving central antitussive, anti-inflammatory, and antioxidant activities. However, most current evidence is derived from *in vitro* studies and animal models, particularly rodents, whereas rigorously designed randomized controlled clinical trials (RCTs) remain almost entirely absent. Specific limitations should be acknowledged. First, there is considerable heterogeneity in dosing regimens, treatment durations, and model construction methods across animal studies, hindering standardized comparison of results. Second, many *in vitro* experiments often use bergenin at concentrations that exceed physiologically relevant levels, potentially leading to overestimation of efficacy. Third, long-term safety data remain scarce. Although traditional use suggests *B. purpurascens* as nontoxic, chronic indications such as diabetes and neurodegenerative diseases would require toxicological studies lasting at least 6–12 months to adequately assess potential cumulative effects and rare adverse events.

Furthermore, *B. purpurascens* is often used in combination with other botanical drugs; however, research on compound prescription mechanisms is still in its early stages. Elucidating metabolite changes associated with compatibility and identifying synergistic mechanisms *in vivo* are key challenges. Current medicinal products are relatively limited, mainly comprising decoction pieces and bergenin tablets, whereas the development of novel bergenin-based drugs is lagging. Consequently, identifying new therapeutic targets and advancing drug development are key to realizing the medicinal potential of *B. purpurascens*.

## 9 Summary and outlook

*Bergenia purpurascens* remains a valuable medicinal resource with considerable potential for development. Integrating its long history of traditional use with modern scientific research may provide new therapeutic options for respiratory, inflammatory, and metabolic diseases.

Despite these advances, several structural limitations in the current body of knowledge should be acknowledged. Regarding resource sustainability, medicinal materials rely primarily on wild harvesting. Habitat destruction and overexploitation, particularly within the China–Himalaya biodiversity hotspot, have threatened its genetic diversity. Although tissue culture and GAP-based cultivation systems have been developed, challenges such as germplasm degradation and prolonged cultivation cycles limit their near-term replacement of wild resources. In cases where bergenin is the sole target compound for extraction, alternative botanical sources such as *R. sambucifolia* may also be considered as potential raw materials. Regarding quality control, the current standards rely almost exclusively on bergenin as the sole quality marker, despite significant chemotypic variations in arbutin, gallic acid, and other phenolics across different producing regions, harvest seasons, and plant parts. A single-marker approach inadequately reflects overall efficacy, necessitating a multicomponent quality control evaluation framework. Regarding pharmacological evidence, the antitussive, expectorant, and anti-inflammatory effects are relatively well-established, with increasingly clear mechanisms. However, studies investigating hepatoprotective, neuroprotective, and antitumor activities remain fragmented, often relying on single cell lines or acute animal models and lacking systematic dose–response analyses or positive-control comparisons. Moreover, RCTs designed rigorously are almost entirely absent, substantially limiting clinical translation. Regarding safety, available toxicological data are largely restricted to acute toxicity assessments, whereas chronic (≥6 months) and reproductive toxicity studies are lacking, posing a barrier to the development of therapeutic strategies for chronic disease management. Regarding compound prescriptions, although frequently used in combinations, the *in vivo* metabolism and synergistic mechanisms of formula components remain poorly understood. Regarding product development, current dosage forms are largely limited to decoction pieces and bergenin tablets, whereas pipelines for lead compound–based novel drugs remain inadequate.

Therefore, future research should prioritize (1) advances in artificial cultivation methods to reduce dependence on wild resources, (2) the development of multicomponent quality control systems integrated with chromatographic fingerprinting, (3) long-term toxicological studies and rigorous RCTs to provide high-level clinical evidence, (4) the application of multiomics and systems pharmacology approaches to elucidate the mechanisms of compound prescriptions, and (5) leveraging synthetic biology advances to drive bergenin-based drug innovation. Through these coordinated efforts, the medicinal potential of *B. purpurascens* can be realized for sustainable human health benefits.
